# Tuberculosis in Pregnancy

**DOI:** 10.1155/S1064744996000208

**Published:** 1996

**Authors:** Kecia Gaither, Joseph J. Apuzzio

**Affiliations:** Department of Obstetrics and Gynecology UMDNJ-New Jersey Medical School 185 South Orange Avenue Newark NJ 07103-2714 USA

## Abstract

Tuberculosis (TB) during pregnancy and in the perinatal period was once considered to be an infrequent event in the United States. After a decade of steady decline, however, the disease has begun a resurgence. According to the CDC, a 20% increase in the number of reported cases occurred between 1985 and 1992. The factors associated with this increase are the emergence of human immunodeficiency virus (HIV) infection, the development of drug-resistant organisms, substance abuse, homelessness, and immigration. Environmental factors promoting transmission can be found in overcrowded areas such as correctional facilities, nursing homes, hospitals, and migrant-worker camps. For a large number of medically underserved women, the obstetrician is the only interface with medical care, as most of these patients do not have primary-care providers. It is important, therefore, that health-care providers recognize the clinical symptoms of TB and follow the recognized guidelines for antenatal screening for TB because the omission of these steps can lead to potentially disastrous sequelae in the fetus and neonate.


Infectious Diseases in Obstetrics and Gynecology 4:92–96 (1996)
© 1996 Wiley-Liss, Inc.

### Tuberculosis in Pregnancy

**Kecia Gaither and Joseph J. Apuzzio**

*Department of Obstetrics and Gynecology, UMDNJ-New Jersey Medical School, Newark, NJ*

### Abstract

**Tuberculosis (TB) during pregnancy and in the perinatal period was once considered to be an infrequent event in the United States. After a decade of steady decline, however, the disease has begun a resurgence. According to the CDC, a 20% increase in the number of reported cases occurred between 1985 and 1992. The factors associated with this increase are the emergence of human immunodeficiency virus (HIV) infection, the development of drug-resistant organisms, substance abuse, homelessness, and immigration. Environmental factors promoting transmission can be found in overcrowded areas such as correctional facilities, nursing homes, hospitals, and migrant-worker camps. For a large number of medically underserved women, the obstetrician is the only interface with medical care, as most of these patients do not have primary-care providers. It is important, therefore, that health-care providers recognize the clinical symptoms of TB and follow the recognized guidelines for antenatal screening for TB because the omission of these steps can lead to potentially disastrous sequelae in the fetus and neonate.** © 1996 Wiley-Liss, Inc.

Key words

*Mycobacterium tuberculosis*, HIV, antituberculosis medications, congenital tuberculosis

Tuberculosis (TB) is one of the most common chronic infectious diseases worldwide. Annually, approximately 7 million new cases are diagnosed, with 2 million deaths directly attributable to the disease. In the United States, from the beginning of the century to 1985, a decline from 100,000 to 20,000 new cases was noted.[1,2] From 1985 to 1992, however, a marked increase in new cases occurred. Several factors account for this rise including the increasing rate of immigrants from endemic areas of the world such as Asia, Africa, and Latin America; the burgeoning rates of homelessness and poverty; the emergence of resistant organisms; and, most importantly, the emergence of acquired immunodeficiency syndrome (AIDS).

At all times, the poor have been a major group affected by TB. The disease tends to occur in clusters or nests based on racial or ethnic minority groups with low socioeconomic status.[3] An overview of deaths from TB in 1985 revealed the mortality rate for nonwhites to be 3.7 times the rate for whites.

The age distribution also demonstrates a marked difference in the rates of disease between minorities and whites. Approximately 40% of cases among nonwhites occur prior to the age of 35 years, while, in whites, the majority of cases occur around the age of 65 years.

Since the prevalence of TB affects a large number of young adults, the same age group constitutes the largest number of patients infected with human immunodeficiency virus (HIV), specifically, the 15–49-year-old age group. The interaction between these 2 diseases poses a 2-fold problem: the unusual extrapulmonary manifestations that are more likely to occur in this population present a diagnostic challenge and, with the high prevalence of drug resistance, important considerations must be addressed when selecting therapy.

Address correspondence/reprint requests to Dr. Kecia Gaither, Department of Obstetrics and Gynecology, UMDNJ-New Jersey Medical School, 185 South Orange Avenue, Newark, NJ 07103-2714.

Review Article

Received March 18, 1996
Accepted June 20, 1996

### PATHOGENESIS

The infectious agent of TB, *Mycobacterium tuberculosis*, is the main human pathogen of the genus *Mycobacterium*, followed by *M. leprae*, the causative organism of leprosy. Other members of the genus known to cause human disease are *M. bovis* and the atypical mycobacterium. The former organism was once responsible for disease in humans prior to the pasteurization of milk, while the latter is responsible for the majority of cases of TB in immunosuppressed individuals.

*M. tuberculosis* is spread by airborne transmission in >90% of the reported cases.[4] Droplet nuclei are produced when an individual with active disease coughs, sneezes, speaks, or sings. The manipulation of lesions and processing of tissues or secretions infected with the organism may also produce droplet nuclei. After inhalation, the nuclei pass down the bronchial tree and implant themselves in a respiratory bronchiole or alveolus beyond the mucociliary clearance system. The bacilli subsequently multiply, usually without any initial resistance from the host. Occasionally, the patient experiences fever, cough, and pleuritic chest pain. The organisms are subsequently engulfed by macrophages, where they remain viable and multiply. After the initiation of an infection, the organisms leave the primary focus in the lung and arrive at regional lymph nodes. From there, they may disseminate throughout the body by lymphohematogenous spread. The organs most commonly seeded during this phase are the lung apices, spleen, liver, meninges, bones, and joints. The genitalia and placenta may also be involved.

After 1–2 months, the host develops cell-mediated immunity and hypersensitivity to the tubercle bacillus which is reflected by the development of a positive tuberculin skin-test result. As immunity develops, the primary infection in the lungs and other organs begins to heal through a combination of resolution, fibrosis, and calcification. Although healing occurs, viable bacilli may persist for many years. If the host later becomes immunosuppressed, e.g., HIV infected, these viable bacilli may again become active, leading to the reactivation of pulmonary TB.

Certain disease states, such as diabetes, and medications, such as corticosteroids and other immunosuppressive drugs, reduce the ability of the host to respond to the organism. An HIV infection, because of its profound immunosuppression, predisposes an individual to more severe forms of TB. In such a patient, dissemination of the bacilli, with unusual clinical manifestations of extrapulmonary disease, is common.

It is important that the definition of the 2 phases of infection be clarified: ***Tuberculosis infection*** is the preclinical stage of *M. tuberculosis;* ***Tuberculosis disease*** occurs when there are clinical manifestations of pulmonary or extrapulmonary involvement and a positive chest X-ray.

### CLINICAL FINDINGS OF ACTIVE DISEASE

Characteristically, in pulmonary TB, there is an onset of cough, which typically progresses over weeks to months. The cough subsequently becomes more frequent, with the production of mucoid or mucopurulent sputum. Hemoptysis may also occur. A recurring, dull, aching pain or chest tightness is common. Dyspnea is uncommon unless massive lung parenchymal involvement occurs. Some patients present with the acute onset of productive cough, fever, chills, myalgias, and sweating, similar to the signs and symptoms of influenza, acute bronchitis, or pneumonia. The physical findings may include rales or signs of lung consolidation, which are generally unilateral.

### SCREENING AND DIAGNOSIS

It is important that all pregnant women at the first prenatal visit be questioned about current symptoms compatible with TB; a previously positive tuberculin test; bacille Calmette-Guérin (BCG) vaccination; previous treatment; membership in a high-risk group; or employment in a hospital, nursing home, or prison. Although any of these situations is sufficient reason for a tuberculin skin test, most physicians feel that all pregnant women should be skin tested.

The tuberculin skin test is based on the fact that an infection with *M. tuberculosis* produces sensitivity to certain antigenic components of the organism. The purified protein derivative (PPD) test is the best means of detecting an infection with *M. tuberculosis*. Furthermore, the patterns of reaction are important in establishing follow-up and preventive therapy with antituberculous agents.

One-tenth of a milliliter of PPD is injected subcutaneously into either the volar or the dorsal surface of the forearm. A wheal of 6–10 mm should be produced if the injection is given correctly. The test should be read between 48 and 72 h later by an experienced health-care provider. The presence or absence of induration is the basis of the reading. For a woman who is at high risk for HIV or who is HIV positive, a delayed-type hypersensitivity anergy test should be employed. Typically, the antigens used are those for mumps, candida, or *Trichophyton*. The diameter of induration should be measured transversely to the long axis of the forearm and recorded in millimeters. According to the American Thoracic Society, a reaction of >5 mm is classified as positive for patients in the following groups: patients with HIV or patients with risk factors and an unknown status; patients with close, recent contact with infectious TB cases; and patients with chest X-rays consistent with old (healed) TB.[5] A reaction of >10 mm is classified as positive in persons not meeting the above criteria who have other risk factors for TB, including the following groups: foreign-born persons, particularly from Asia, Africa, and Latin America; patients with preexisting medical conditions such as diabetes mellitus or malignancies that place them at increased risk; medically underserved, low-income populations; and health-care workers.

There exists no definitive method of distinguishing a tuberculin reaction caused by a natural mycobacterial infection from a reaction caused by a BCG vaccination. Generally, a reaction size of >10 mm is considered positive for a patient who has previously received BCG, particularly if the immunization occurred more than a decade previously. Among the multiple reasons for not assuming that a large reaction is secondary to BCG are that tuberculin sensitivity tends to wane after vaccination and the mean reaction size among persons vaccinated is often <10 mm. As many BCG-vaccinated individuals come from areas of the world where TB is endemic, an individual with a significant reaction to tuberculin skin testing should be further evaluated for the presence of disease and managed accordingly.

### SPECIMEN COLLECTION

Because the identification of mycobacteria is important in diagnosing TB, the proper collection and handling of specimens are imperative. A culture of the organism is mandatory for a definitive diagnosis of the disease. *M. tuberculosis* is an obligate aerobe that is slow growing in classic culture media, often taking weeks for identification. Recently, however, a rapid radiometric method, the BACTEC method (BACTEC, Johnston Laboratories, Towson, MD) has become available for the detection and differentiation of mycobacteria and drug-susceptibility testing.[6] The BACTEC technique permits a rapid diagnosis in days compared with weeks or even months with the classic culture media.

Although radiometric technology cannot completely replace the standard mycobacteriologic culture methods and may underestimate drug resistance, it is a valuable new tool. Other methods for microbiologic identification include genetic probes, enzyme-linked immunosorbent assays (ELISA), mycobacteriophage typing, and high-performance liquid chromatography (HPLC) to detect species-specific mycolic acids.[5]

Since tuberculous disease can occur in almost any site in the body, a variety of materials may be substituted for collection. In addition to the most common specimens such as sputum and gastric aspirate, urine, cerebrospinal fluid, pus, and bronchial washings can be submitted. A placental examination can lead to detection in the mother.[7]

### TB IN PREGNANCY

Throughout history, medical opinion concerning the effect of pregnancy on the course of TB has varied. Prior to the 18th century, pregnancy was viewed as having a beneficial effect. In contrast, the opinion held nearly a century later was that pregnancy imposed a detrimental effect on the course of TB justifying the recommendation for a therapeutic abortion. Pregnancy does not appear to be a risk factor for the development of TB, nor does it accelerate the clinical course of the disease—even in HIV-infected women.[8,9] TB is seldom an indication for an abortion unless massive dissemination or severely compromised cardiopulmonary function occurs.[10] Problems in managing TB in pregnant patients are usually not due to any potential maternal respiratory impairment, but to the possible teratogenic effects of antituberculous medication.

During pregnancy, a positive tuberculin test is evaluated by a chest X-ray with abdominal shielding of the gravid uterus. Radiation exposure to the fetus with proper shielding has been estimated to be <0.3 millirad.[11,12] A woman with a suspicious chest X-ray for active disease should undergo 3 early morning sputum samples for culture and smear.

A pregnant woman with a positive PPD, without evidence of active disease on her chest X-ray, should undergo isoniazid prophylaxis. The prophylactic regimen consists of isoniazid for 6–12 months starting in the postpartum period.

A woman with HIV infection or risk factors who refuses testing should also be treated prophylactically for a positive PPD. For such a woman, 4 months of isoniazid and rifampin or 12 months of isoniazid is recommended, providing that drug-resistant organisms are unlikely.[13] In a pregnant patient, however, who has a recent conversion (within the last 2 years) or close contact with someone with active disease, prophylaxis is initiated after the first trimester.

In contrast to this recommendation, a patient with active disease should begin chemoprophylaxis immediately regardless of the gestational age. Untreated TB poses a greater hazard to the pregnant woman and her fetus than the treatment of the disease does. Once sputum cultures have been obtained to confirm the diagnosis with indicated susceptibilities, the pregnant woman with TB should receive multidrug chemotherapy for 9 months. The treatment of the disease consists of isoniazid, rifampin, and ethambutol. Vitamin B6 is indicated for all pregnant women taking isoniazid. Although pyrazinamide has been used abroad for the treatment of disease, it is not used during pregnancy in the United States because of concerns of potential teratogenicity.[14]

### ANTITUBERCULOUS MEDICATIONS

Isoniazid, ethambutol, and rifampin have all been evaluated for potential fetal effects during pregnancy. To date, none has been found to be teratogenic. All 3 drugs are bactericidal. Furthermore, all 3 cross the placenta as well as appear in the breast milk.

Isoniazid is the most widely used chemotherapeutic drug for TB. It is easily administered and inexpensive. It can be given orally or by intramuscular injection. The recommended dose is 300 mg/day. Although it is excreted in breast milk, its use during the postpartum period does not contraindicate breast-feeding so long as there is adequate pediatric follow-up. The potential side effects of isoniazid include hepatitis and peripheral neuropathy. It is useful to obtain baseline serum transaminase levels at the beginning of therapy and monitor the patient clinically for adverse reactions during the course of pregnancy. As isoniazid interferes with the metabolism of pyridoxine, causing peripheral neuritis, 50 mg/day of vitamin B6 is recommended.

The usual dose of ethambutol is 15 mg/kg. Retrobulbar neuritis is the most serious side effect, the symptoms including red-green color blindness, blurred vision, and central scotomata. The ocular effects are dose related and generally noted in patients with renal insufficiency.

The recommended dosage of rifampin is 10 mg/kg, with a usual daily dosage of 600 mg/day. The most common adverse reaction is gastrointestinal upset.

### CONGENITAL TB

Congenital TB is extremely rare, with <200 cases reported in the literature.[15] Neonatal TB can either be truly congenital (acquired in utero) or truly neonatal (acquired in early life from an infected mother or caretaker). Congenital TB may be acquired through 3 routes: 1) from the infected placenta through hematogenous dissemination from the umbilical vein, 2) by inhalation of infected amniotic fluid, or 3) by ingestion of infected amniotic fluid.

In hematogenously acquired congenital TB, the organisms reach the fetus through the umbilical vein. Generally, a primary focus develops in the fetal liver, with associated involvement of the periportal lymph nodes. However, the bacilli can also pass through the liver into the main circulation, leading to a primary focus in the lung where it remains dormant until birth.

Inhalation or ingestion of infected amniotic fluid by the fetus results from caseous placental lesions rupturing into the amniotic cavity. In this instance, multiple foci are present in the lung, gut, and middle ear.

Postnatal TB can be acquired in 4 different ways: 1) by inhalation of infected droplets, 2) by ingestion of infected droplets, 3) by ingestion of infected milk, or 4) by contamination of traumatized skin or mucous membranes. Because newborns infected with TB are at extremely high risk of developing severe forms of the disease, the investigation of a person with TB whose household contains a pregnant woman or newborn infant should be considered a public-health emergency.

### CONCLUSIONS

With the increase in reported cases of TB along with the spread of drug-resistant strains, heightened vigilance concerning recognition, prevention, and treatment has begun. The obstetric patient with active disease not only exposes healthy individuals to the organism, but her fetus as well. With appropriate history-taking and screening, TB can be diagnosed and treated successfully. A woman's pre- and post-delivery care represents the best opportunity for such an outcome.

### REFERENCES

1. Centers for Disease Control and Prevention: Initial therapy for tuberculosis in the era of multidrug resistance: Recommendations of the advisory council for the elimination of tuberculosis. MMWR 42:7, 1993.
2. Centers for Disease Control and Prevention: Tuberculosis Statistics: States and Cities. Atlanta: CDC, 1985.
3. La Force FM, Huber GL, Fahey JM: The focality of urban tuberculosis. A look at Boston and its south end. Am Rev Respir Dis 108:553–558, 1978.
4. Maccato ML: Pneumonia and pulmonary TB in pregnancy. Obstet Gynecol Clin N Am 16(2):417–430, 1989.
5. American Thoracic Society: Diagnostic standards and classification of tuberculosis. Am Rev Respir Dis 142:725–735, 1990.
6. Siddiqi SH, Hwangbo CC, Silcox V, Good RC, Snider DEJ, Middlebrook G: Rapid radiometric methods to detect and differentiate *Mycobacterium tuberculosis/M. bovis* from other mycobacterial species. Am Rev Respir Dis 130:634–640, 1984.
7. Kaplan C, Benirschke K, Tarzy B: Placental tuberculosis in early and late pregnancy. Am J Obstet Gynecol 137:858–860, 1980.
8. Snider D: Pregnancy and tuberculosis. Chest 86:105–135, 1984.
9. Morgano R, Mroueh J, Garely A, White D, Duerr A, Minkoff H: Resurgence of active tuberculosis among pregnant women. Obstet Gynecol 83:911–914, 1994.
10. Swartz HM: Radiation risk from chest examination. JAMA 239:1907, 1978.
11. Brent R, Garson RO: Radiation exposure in pregnancy. Curr Probl Radiol 2:3–48, 1972.
12. Ad Hoc Committee of the Scientific Assembly on Microbiology, Tuberculosis and Pulmonary Infections: Treatment of tuberculosis and tuberculous infection in adults and children. Clin Infect Dis 21:9–27, 1995.
13. Smith MHD, Teele DW: Tuberculosis—Infectious Disease of the Fetus and Newborn. 3rd ed. Philadelphia: W.B. Saunders, pp 834–847, 1990.
14. Vallejo JG, Starke J: Tuberculosis and pregnancy. Clin Chest Med 13(4): 693–707, 1992.
15. Sudre P, Dam G ten, Kochi A: Tuberculosis: A global overview of the situation today. Bull WHO 70(2):149–159, 1992.
